# Determination of Lipophilic Marine Biotoxins in Mussels Harvested from the Adriatic Sea by LC-MS/MS

**DOI:** 10.3389/fmicb.2018.00152

**Published:** 2018-02-12

**Authors:** Maria Schirone, Miriam Berti, Pierina Visciano, Francesco Chiumiento, Giacomo Migliorati, Rosanna Tofalo, Giovanna Suzzi, Federica Di Giacinto, Nicola Ferri

**Affiliations:** ^1^Faculty of Bioscience and Technology for Food, Agriculture and Environment, University of Teramo, Teramo, Italy; ^2^Biologia delle Acque Interne, Istituto Zooprofilattico Sperimentale dell'Abruzzo e del Molise “G. Caporale”, Teramo, Italy; ^3^Bromatologia e Residui negli Alimenti per l'Uomo e gli Animali, Istituto Zooprofilattico Sperimentale dell'Abruzzo e del Molise “G. Caporale”, Teramo, Italy

**Keywords:** marine biotoxins, okadaic acid, dinophysistoxin, azaspiracid, yessotoxin, *Mytilus galloprovincialis*, LC-MS/MS

## Abstract

Lipophilic marine biotoxins include okadaic acid, pectenotoxin, yessotoxin and azaspiracid groups. The consumption of contaminated molluscs can lead to acute food poisoning syndromes depending on the exposure level. Regulatory limits have been set by Regulation (European Community, 2004a) No 853/2004 and LC-MS/MS is used as the official analytical method according to Regulation (European Community, 2011) No 15/2011. In this study specimens of mussels (*Mytilus galloprovincialis*) were collected along the coasts of the central Adriatic Sea during the years 2015–2017 and analyzed by the European harmonized Standard Operating Procedure. The method was validated for linearity, specificity, repeatability and reproducibility and it revealed able to be used for the detection of the lipophilic marine biotoxins. Levels of okadaic acid, pectenotoxin, yessotoxin and its analogs were detected at different concentrations in 148 (37%) out of a total of 400 samples, always below the maximum limits, except for 11 (4.3%) of them that were non-compliant because they exceeded the regulatory limit. Moreover, some samples were exposed to a multi-toxin mixture with regards to okadaic acid, yessotoxin and 1-Homo yessotoxin. Following these results, the aquaculture farms from which the non-compliant samples derived were closed until the analytical data of two consecutive samplings returned favorable. Besides the potential risk of consumption of mussels contaminated by lipophilic marine biotoxins, these marine organisms can be considered as bio-indicators of the contamination status of the marine ecosystem.

## Introduction

Lipophilic marine biotoxins (LMB) are toxic metabolites produced by some species of unicellular algae developing during natural phenomena known as harmful algal blooms. They are grouped in different classes, i.e., okadaic acid (OA), azaspiracids (AZA), yessotoxins (YTX), pectenotoxins (PTX), and spirolides (Ferron et al., 2016).

The OA group consists of OA and its isomers, the dinophysistoxins1 and 2 (DTX1, DTX2) and in addition the fatty acid ester derivatives of OA or DTX1 and DTX2 named DTX3 (Braga et al., 2017). These marine biotoxins are responsible of the human diarrhetic shellfish poisoning (DSP) characterized by gastrointestinal disorders such as nausea, vomiting, severe diarrhea and abdominal cramps (García et al., 2016). The mechanism of action of OA and DTX is linked to the inhibition of serine/threonine protein phosphatases (Ferreiro et al., 2015).

Besides gastrointestinal symptoms, AZA can be carcinogenic in mice and teratogenic to the developing fish (Twiner et al., 2012). Moreover, azaspiracid1 (AZA1) was demonstrated to be toxic to some human cell lines (B lymphocyte, monocyte, lung epithelial, T lymphocyte); azaspiracid2 (AZA2) has been shown to have a similar cytotoxicity with cytoskeleton alterations (Kim et al., 2017). Ferreiro et al. (2014a,b) reported that AZA2 could have acute arrhythmogenic potential *in vivo* and chronic effects on a specific cardiac potassium channel *in vitro*. Also YTX and its analogs can present cardiotoxicity with mitochondrial damage in cardiomyocytes after repeated exposure and marked bradycardia and hypotension in rats (Ferreiro et al., 2016), while some PTX are hepatotoxic to mice by intraperitoneal injection (Trainer et al., 2013). The European Legislation set maximum levels in Regulations (European Community, 2004a) No 853/2004 and (European Community, 2013) No 786/2013, corresponding to 160 μg of OA equivalent kg^−1^ for OA, DTX and PTX together, 160 μg of AZA equivalent kg^−1^ for AZA and 3.75 mg YTX equivalent kg^−1^ for YTX-group.

The Regulation (European Community, 2005) No 2074/2005 established the official analytical methods to be used for the detection of LMB, which were represented by the mouse bioassay (MBA) and the rat bioassay (RBA). However, these methods showed some disadvantages other than ethical, such as the high variability in results, the insufficient detection capability and the limited specificity. Therefore, developed alternatives to the biological methods were successfully tested and a liquid chromatography-mass spectrometry (LC-MS/MS) method was validated and recognized as the official method by the Regulation (European Community, 2011) No 15/2011 since 31 December 2014. The aim of this study was the application of the EU Harmonised Standard Operating Procedure for determination of Lipophilic marine biotoxins in molluscs by LC-MS/MS (2015) in specimens of *Mytilus galloprovincialis* coming from different aquaculture farms located along the central Adriatic coasts. The samples were collected according to the multi-annual regional control plan 2015–2018, which requires the samplings twice a month. Another objective of this study was the validation of the method for the criteria established by Regulation (European Community, 2004c) No 882/2004.

## Materials and methods

### Collection of specimens and sample preparation

**Figure 1:**
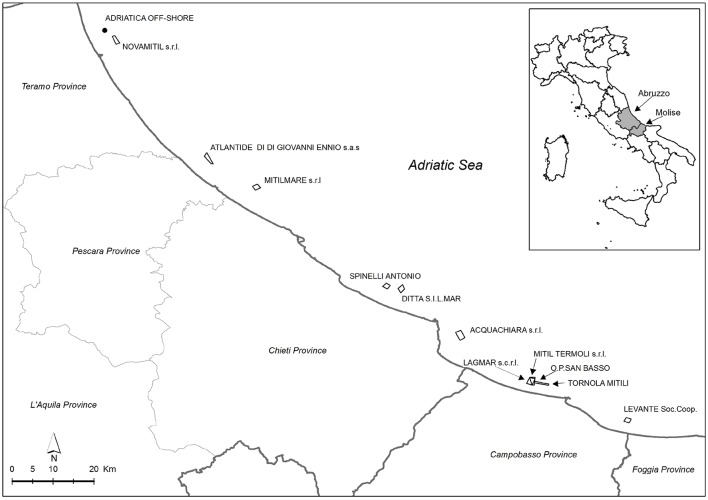
Map of the sampling sites along the coasts of the central Adriatic Sea.

Specimens of *M. galloprovincialis* were collected from 12 aquaculture farms located along the coasts of Abruzzo and Molise regions, in coastal areas belonging to the following 4 provinces: Teramo, Pescara, Chieti (Abruzzo) and Campobasso (Molise). The samples were taken from 4 different sampling points (from A to D), except for a farm in which just only one sampling point was considered (Figure 1). In Table 1, longitude and latitude for each sampling point were reported.

**Table 1:** Longitude and latitude of the sampling points.

**Region**	**Aquaculture Farm**	**Points**	**Longitude (East)**	**Latitude (Nord)**
Abruzzo	Adriatica Off-Shore	A	13,985277	42,808611
	Novamitil S.R.L.	A-D	14,001666/14,006667/14,018334/14,009999	42,794999/42,797499/42,780833/42,778331
	Atlantide Di Giovanni	A-D	14,204166/14,209722/14,223611/14,218055	42,533333/42,541666/42,516666/42,518055
	Mitilmare S.R.L.	A-D	14,310097/14,321057/14,327828/14,316638	42,467663/42,472476/42,464385/42,459421
	Spinelli Antonio	A-D	14,596670/14,604170/14,613330/14,606670	42,248670/42,256170/42,251000/42,243500
	Ditta S.I.L. Mar	A-D	14,630555/14,640277/14,644444/14,636111	42,244444/42,252777/42,243055/42,234722
	Acquachiara S.R.L.	A-D	14,756833/14,768333/14,777130/14,766667	42,148167/42,152667/42,137642/42,131167
Molise	Lagmar S.C.R.L.	A-D	14,920000/14,931667/14,925000/14,913333	42,050000/42,046670/42,033330/42,036670
	Mitil Termoli S.R.L.	A-D	14,920000/14,931944/14,930000/14,925000	42,050000/42,050000/42,033330/42,033330
	O.P. San Basso	A-D	14,931110/14,947500/14,946944/14,930000	42,041390/42,038610/42,035000/42,038060
	Tornola Mitili	A-D	14,949157/14,962088/14,961113/14,948183	42,037970/42,035570/42,032620/42,034900
	Levante Soc. Coop.	A-D	15,132375/15,143406/15,138489/15,127458	41,962340/41,958650/41,950450/41,954140

The sampling was made according to the multi-annual regional control plan 2015–2018. The frequency for the determination of marine biotoxins was established twice in a month or more frequently when the contamination in mussels increased.

A total of 400 global samples, each formed by 150 specimens of *M*. *galloprovincialis*, were collected during the investigated years, and more in detail 126 samples in 2015, 173 samples in 2016, and 101 samples from January to July 2017.

The mussels were opened, removed from the shell, washed with running water to remove any residues and pooled according to their origin, to form the global samples. Then, each global sample was homogenized with a blender and stored at −20°C until the analysis. The extraction procedure was carried out for 2 g of the homogenate.

### Chemicals and standards preparation

All chemicals were of analytical reagent grade: methanol (LC-MS grade), acetonitrile (LC-MS grade) and ammonium formate (99% purity), formic acid (98%), hydrochloric acid (37%), and sodium hydroxide (99%) and were purchased from Sigma–Aldrich (St. Louis, MO, USA). Water was prepared with high-purity water obtained from a Milli-Q® system (Merck Millipore, Darmstadt, Germany).

The certified reference standard solutions of OA (CRM-OA-c), DTX1 (CRM-DTX1) and DTX2 (CRM-DTX2), PTX2 (CRM-PTX2), AZA1 (CRM-AZA1), AZA2 (CRM-AZA2), AZA3 (CRM-AZA3), YTX (CRM-YTX), 1-Homo YTX (CRM-Homo YTX), certified reference material with OA, and DTX (CRM-DSP-MUS-b), homogenate of digestive gland of mussel (*Mytilus edulis*) with OA and DTX were all purchased from the Certified Reference Materials Program of the Institute for Marine Biosciences, National Research Council Canada (Ottawa, Ontario, Canada). The calibration curve for all biotoxins was prepared in methanol following the EU-harmonized SOP.

### Instrumental analysis

The instrumental analysis was performed by a Perkin Elmer HPLC system (Perkin Elmer, Waltham, MA, USA) constituted of a model 200 microbinary pump and a model 200 auto-sampler. A reversed-phase HPLC column X-Bridge C18 (50 × 2.1 mm, 2.5 μm) with relative guard column X-Bridge C18 (10 × 2.1 mm, 2.5 μm) both from Waters (Milford, MA, USA) were used. The flow rate was set to 0.3 ml min^−1^ and the injection volume was 20 μl. The mobile phase was used in gradient mode as follows: 90% of eluent A (100% water containing 2 mM ammonium formate and 50 mM formic acid) and 10% of eluent B (95% acetonitrile: 5% water containing 2 mM ammonium formate and 50 mM formic acid) at 0–10 min, then eluent B increased up to 90% at time 10–13 min and decreased again to 10% at 13–16 min up to 20 min.

The LC-MS/MS analysis was carried out by a mass spectrometer API 3000 PE SCIEX (Applied Biosystems, Toronto, ON, Canada) equipped with an electrospray interface set in the positive ionization mode (ESI+) for PTX and AZA-groups, and the negative ionization mode (ESI−) for OA, DTX- and YTX-groups. The mass spectrometer was set in multiple reaction monitoring (MRM) mode, with specific transition parameters as reported in Table 2. The capillary voltage was set at 5.5 kV for ESI+, −4.5 kV for ESI−, and the ion source temperature at 550°C.

**Table 2:** LC-MS/MS parameters for detection of marine biotoxins in MRM mode.

**Biotoxin**	**ESI**	**Precursor ion ⇒ product ion (*m/z*)**	**DP^a^**	**FP^b^**	**EP^c^**	**CE^d^**	**CXP^e^**
OA	N^1^	803.6 ⇒ 255.3 803.6 ⇒ 113.3	−146	−350	−10	−65 −78	−4
DTX1	N	817.6 ⇒ 255.5 817.6 ⇒ 112.8	−130	−340	−13	−90 −70	−10
DTX2	N	803.6 ⇒ 255.3 803.6 ⇒ 113.3	−80	−315	−13	−65 −88	−1 −6
YTX	N	1,141.6 ⇒ 1,061.7 1,141.6 ⇒ 855.6	−110	−350	−14	−45 −96	−16 −10
1-Homo YTX	N	1,155.6 ⇒ 1,075.6 1,155.6 ⇒ 869.6	−100	−350	−14	−46 −100	−18 −10
45 OH YTX	N	1,157.5 ⇒ 1,077.7 1,157.5 ⇒ 871.5	−110	−350	−14	−45 −96	−16 −10
45 OH-Homo YTX	N	1,171.5 ⇒ 1,091.5 1,171.5 ⇒ 869.9	−100	−350	−14	−46 −100	−18 −10
AZA1	P^2^	842.4 ⇒ 824.4 842.6 ⇒ 806.7	85	385	10	40 55	10 11
AZA2	P	856.6 ⇒ 838.5 856.6 ⇒ 820.6	81	360	10	43 53	12
AZA3	P	828.6 ⇒ 810.6 828.6 ⇒ 792.4	72	360	8	43 53	18 11
PTX2	P	876.6 ⇒ 823.5 876.6 ⇒ 212.9	74	375	9	33 36	11
PTX1	P	892.5 ⇒ 821.5 892.5 ⇒ 213.2	74	375	9	33 36	11

^1^N, Negative; ^2^P, Positive;

a DP, Declustering Potential;

b FP, Focusing Potential;

c EP, Entrance Potential;

d CE, Collision Energy;

e *CXP, Collision Cell Exit Potential*.

## Results and discussion

### Concentrations of lipophilic marine biotoxins

In this study, the monitoring for LMB occurrence in specimens of *M*. *galloprovincialis* collected during the years 2015–2017 showed a good trend with regards to the compliance with the regulatory limits. To express the results by each toxin group according to the European legislation (i.e., as μg equivalents kg^−1^ or mg equivalents kg^−1^) the use of the Toxicity Equivalent Factors (TEFs) was required. Therefore, the individual content of each detected biotoxin/analog was multiplied with the corresponding TEF before summarizing the total equivalents for the respective group toxins (European Food Safety Authority, 2009).

Among the investigated LMB, concentrations above the LOQ were found only for OA, PTX, YTX, and its analogs. Moreover, mussels were often exposed to a multi-toxin mixture because some samples contained more than one LMB. The simultaneous presence of OA, YTX, and 1-Homo YTX were detected in 17 and 11 samples collected in 2015 and 2016 years, respectively. Moreover, 6 out of these 28 samples had also PTX2 concentrations. A total of 11 samples exceeded the regulatory maximum limit of 160 μg of OA equivalents kg^−1^ (Table 3). The samples showing only OA levels ranging from 42.5 to 114 μg of OA equivalents kg^−1^ were reported in Table 4. The sample named V6, collected from Chieti province, had a value of 202 ± 47 μg of OA equivalents kg^−1^, but it was considered compliant based on the measurement uncertainty. Table 5 showed samples containing only YTX and its analogs, even if none of them exceeded the maximum limit for this group. The remaining investigated LMB, i.e., AZA- and DTX- groups, and 45 OH-Homo YTX, were never detected.

**Table 3:** Simultaneous presence of okadaic acid (OA), yessotoxin (YTX), and 1-Homo YTX in samples distinguished by provenance.

**Sample**	**Province**	**Region**	**OA (μg kg^−1^ ± MU)**	**YTX (mg/kg)**	**1-Homo YTX (mg/kg)**	**PTX2 (μg kg^−1^ ± MU)**
**2015**						
TM1	Campobasso	Molise	51.2		0.072	
C1	Chieti	Abruzzo	46.7		0.088	
V1	Chieti	Abruzzo	143	0.172	0.165	
C2	Chieti	Abruzzo	179 ± 42	0.330	0.247	
O1	Chieti	Abruzzo	172 ± 40	0.490	0.434	
TM2	Campobasso	Molise	141	0.121	0.107	
V2	Chieti	Abruzzo	132	0.220	0.196	
P1	Pescara	Abruzzo	120	0.093	0.075	
V3	Chieti	Abruzzo	157	0.183	0.224	
O2	Chieti	Abruzzo	122	0.134	0.153	
O3	Chieti	Abruzzo	111	0.076		
C3	Chieti	Abruzzo	83.7		0.109	
O4	Chieti	Abruzzo	316 ± 74^*^	0.178	0.160	53.2 ± 18.0
C4	Chieti	Abruzzo	758 ± 177^*^	0.199	0.170	93.5 ± 31.6
G1	Teramo	Abruzzo	45.9	0.133	0.107	
TM3	Campobasso	Molise	146	0.167	0.175	
TM4	Campobasso	Molise	409 ± 96^*^	0.179	0.151	75.1 ± 25.4
TM5	Campobasso	Molise	369 ± 86^*^	0.226	0.168	61.7 ± 20.9
P2	Pescara	Abruzzo		0.193	0.203	
C5	Chieti	Abruzzo	179 ± 42	0.244	0.230	
V4	Chieti	Abruzzo	342 ± 80^*^	0.187	0.193	72.6 ± 17.4
F1	Chieti	Abruzzo	523 ± 122^*^	0.144	0.140	
**2016**						
TM6	Campobasso	Molise	265 ± 62^*^	0.215	0.172	
TM7	Campobasso	Molise	139	0.124	0.092	
P3	Pescara	Abruzzo		0.158	0.203	
TM8	Campobasso	Molise	227 ± 53*	0.150	0.071	
V5	Chieti	Abruzzo	168 ± 39	0.226	0.153	
O5	Chieti	Abruzzo	202 ± 47	0.188	0.130	
C6	Chieti	Abruzzo	472 ± 110^*^	0.251	0.214	59.3 ± 14.2
TM9	Campobasso	Molise	55.8	0.104	0.063	
O6	Chieti	Abruzzo	128	0.121	0.113	
C7	Chieti	Abruzzo	132	0.272	0.239	
C8	Chieti	Abruzzo	206 ± 48	0.243	0.145	
TM10	Campobasso	Molise	229 ± 53^*^	0.217	0.124	
C9	Chieti	Abruzzo	214 ± 51^*^	0.091		
TM11	Campobasso	Molise	57.2		0.196	

* *Samples exceeding the maximum limit and non-compliant based on the Measurement Uncertainty (MU)*.

**Table 4:** Concentration of okadaic acid (OA) in samples distinguished by provenance.

**Sample**	**Province**	**Region**	**OA (μg kg^−1^ ± MU)**
**2015**			
O7	Chieti	Abruzzo	57.4
T1	Teramo	Abruzzo	95.9
TM12	Campobasso	Molise	86.7
V6	Chieti	Abruzzo	202 ± 47
**2016**			
TM13	Campobasso	Molise	84.2
TM14	Campobasso	Molise	91.3
TM15	Campobasso	Molise	118
TM16	Campobasso	Molise	72.5
TM17	Campobasso	Molise	103
C10	Chieti	Abruzzo	114
TM18	Campobasso	Molise	42.5
TM19	Campobasso	Molise	44.6
O8	Chieti	Abruzzo	65.6
O9	Chieti	Abruzzo	55.1
C11	Chieti	Abruzzo	45.3
O10	Chieti	Abruzzo	60.0
TM20	Campobasso	Molise	57.2

**Table 5:** Concentrations (mg/kg) of yessotoxin (YTX) and its analogs distinguished for provenance.

**N° of samples**	**Province**	**Region**	**YTX**	**45 OH-YTX**	**1-Homo YTX**
**2015**					
19	Teramo	Abruzzo	0.095 (T2), 0.081 (R1), 0.106 (G3), 0.112 (R2), 0.068 (R3), 0.080 (G9), 0.265 (T3), 0.208 (G10), 0.070 (R4), 1.400 (G11), 0.189 (G12), 0.172 (G13), 0.078 (G14)	0.095 (T2), 0.077 (G3), 0.099 (G5), 0.080 (G6), 0.088 (R2)	0.183 (T2), 0.073 (R1), 0.122 (G2), 0.150 (G3), 0.088 (G4), 0.135 (G6), 0.116 (G7), 0.083 (G8), 0.898 (R2), 0.088 (G9), 0.224 (T3), 0.245 (G10), 1.290 (G11), 0.204 (G12), 0.168 (G13)
8	Pescara	Abruzzo	0.099 (P4), 0.093 (P6), 0.134 (P7), 0.096 (P9), 0.213 (P11)	0.085 (P7)	0.160 (P4), 0.128 (P5), 0.078 (P6), 0.143 (P7), 0.078 (P8), 0.069 (P10), 0.204 (P11)
16	Chieti	Abruzzo	0.067 (V7), 0.088 (V8), 0.099 (F2), 0.110 (V9), 0.084 (F3), 0.132 (C13), 0.210 (V11), 0.092 (F4), 0.121 (O11), 0.183 (V12), 0.172 (O12), 0.165 (C14), 0.126 (FS1), 1.080 (O13)	0.074 (V8), 0.082 (F2), 0.091 (V9)	0.091 (V8), 0.106 (F2), 0.100 (C12), 0.143 (V9), 0.112 (V10), 0.124 (C13), 0.173 (V11), 0.077 (F4), 0.118 (O11), 0.154 (V12), 0.160 (O12), 0.104 (C14), 0.084 (FS1), 1.02 (O13)
14	Campobasso	Molise	0.103 (TM22), 0.093 (TM23), 0.101 (TM25), 0.092 (TM26), 0.200 (TM27), 0.087 (TM28), 0.136 (TM29), 0.176 (TM30), 0.111 (TM31), 0.118 (TM32), 0.105 (TM33)	0.091 (TM23)	0.110 (TM21), 0.131 (TM22), 0.076 (CB1), 0.116 (TM24), 0.095 (TM25), 0.145 (TM27), 0.093 (TM28), 0.115 (TM29), 0.107 (TM30), 0.062 (TM31), 0.089 (TM32), 0.072 (TM33)
**2016**					
1	Teramo	Abruzzo	0.096 (G15)		
1	Pescara	Abruzzo	0.073 (P12)		0.072 (P12)
2	Chieti	Abruzzo	0.109 (C15), 0.094 (V13)		
2	Campobasso	Molise	0.107 (TM34), 0.105 (TM35)		
**2017**					
6	Teramo	Abruzzo	0.133 (T4), 0.108 (T5), 0.090 (T6), 0.167 (T7), 0.186 (T8), 0.232 (T9)		
17	Chieti	Abruzzo	0.229 (O14), 0.397 (V14), 0.286 (O15), 0.156 (V15), 0.257 (C16), 0.245 (C17), 0.167 (C18), 0.137 (V16), 0.522 (C19), 0.215 (C20), 0.145 (O16), 0.282 (O17), 0.153 (C21), 0.184 (V17), 0.125 (O18), 0.162 (C22), 0.173 (C23)		
10	Campobasso	Molise	0.344 (TM36), 0.172 (TM37), 0.238 (TM38), 0.267 (TM39), 0.192 (TM40), 0.142 (TM41), 0.135 (TM42), 0.155 (TM43), 0.144 (TM44), 0.130 (TM45)		

The occurrence of marine harmful algae is increasing worldwide and therefore, the accumulation of LMB from harmful phytoplankton represents a food safety threat in the shellfish industry. In the present study, LMB belonging to the OA-group were the only compounds exceeding the regulatory limits of 160 μg of OA equivalent kg^−1^. In particular, the samples coming from Chieti and Campobasso provinces resulted non-compliant and therefore their production areas were closed according the multi-annual regional control plan 2015–2018 for Abruzzo^1^ and Molise regions. Moreover, this plan established that in case of non-compliant samples, the rapid alert system should be activated and harvesting molluscs from the contaminated areas should be suspended until the results of two consecutive samplings are compliant. In addition, Regulation (European Community, 2004b) No 854/2004 affirms that in such circumstance the production area of bivalve molluscs should be closed by the competent authority and it could be re-opened when at least two consecutive results of biotoxin levels meet with the legislative criteria.

Also other authors reported frequent closures of bivalve fisheries of the west coast of Ireland due to AZA presence in the blue edible mussel *M. edulis* (Murray et al., 2017).

Our previous studies carried out in mussels coming from the central Adriatic Sea showed a similar trend with regards to YTX content, while no presence of the other investigated LMB was observed. The monitoring plans for the determination of LMB and domoic acid (DA) in samples of *M*. *galloprovincialis* during the years 2006–2009 revealed no presence of these compounds, neither by MBA used for LMB, nor by a chromatographic analysis for DA detection (Schirone et al., 2011). These results demonstrated a good condition of the monitored marine zones for the occurrence of marine biotoxins, even if a high intensity of algal blooms in Adriatic Sea have been frequently reported in the last years. However, the DSP outbreak occurrence remains still very difficult to predict due to the high variability of biotoxin content in phytoplankton cells (Leonardo et al., 2017). In another study (Schirone et al., 2013), YTX levels were found in *M*. *galloprovincialis* specimens taken from three Italian regions (i.e., Abruzzo, Molise, Emilia Romagna) along the coasts of the Adriatic Sea, at concentrations ranging from 0.2 to 1.8 mg of YTX equivalent kg^−1^. Some samples coming from Emilia Romagna region exceeded the maximum limit (1 mg of YTX equivalent kg^−1^) that was in force in the period of the investigation, instead of the new limit of 3.75 mg of YTX equivalent kg^−1^ fixed by Regulation (European Community, 2013) No 786/2013. These analyses were carried out by a functional method as alternative to MBA for the *in vitro* quantitative detection of YTX. The positive samples were also confirmed by MBA causing the death of two out three mice within 24 h of inoculation with the extract of the mollusc, even if this method showed low specificity and sensitivity because it did not provide the identification and quantification of the biotoxin causing the death of mice. A comparison among the two cited assays and a LC-MS/MS method was studied by analyzing other samples of *M*. *galloprovincialis* collected from the Adriatic Sea (Visciano et al., 2013). The results showed the presence of YTX at concentrations up to 1.63 and 1.97 mg of YTX equivalent kg^−1^ by the functional assay and LC-MS/MS, respectively. Moreover, the last method allowed the detection also of YTX analogs, i.e., homo YTX and carboxy homo YTX. The authors supposed that the influx of the waters from the Po, the most important river of Italy, on phytoplankton bloom dynamics, as well as the clear seasonal variability in the circulation and ecosystem of the Adriatic Sea due to strong forcing functions could affect the presence of this compound.

In the present study the concentrations of YTX and its analogs were lower than the above reported values (Schirone et al., 2013; Visciano et al., 2013) while levels of LMB belonging to OA-group were detected for the first time. These results confirmed that OA is gaining a high critical interest because it represents the most predominant DSP biotoxin in the European coasts (González-Romero et al., 2012). Bacchiocchi et al. (2015) found concentrations ranging between 5 and 25 μg of OA equivalent kg^−1^ in samples of *M. galloprovincialis* collected along the coast of Marche region, Adriatic Sea (Italy) during the years 2012–2013, whereas DTX were never detected. Levels of YTX and its analogs were also reported, reaching values near or slightly above the regulatory limit. However, Pistocchi et al. (2012) described that the Northern Adriatic Sea has been characterized by the presence of toxic algae producing OA and DTX until 1997 and YTX in the past decades. Moreover, Orellana et al. (2017) found similar results to those obtained in the present study, showing that OA/DTX2 and YTX were the most abundantly accumulated LMB in the analyzed mussels (*M. edulis, Crassostrea gigas* and *Patella* sp.) coming from the Belgian Part of the North Sea and reaching values of 25.4 and 169.2 μg equivalent kg^−1^ wet, respectively. Gerssen et al. (2010) found concentrations ranging from 18.2 to 67.5 μg of OA equivalent kg^−1^ in mussels (*M. edulis*) collected from the Dutch shellfish harvesting areas.

Levels of OA, ranging from 40 to 611 μg of OA equivalent kg^−1^ were also detected by Garibo et al. (2014) in samples of *M*. *galloprovincialis* obtained from the shellfish monitoring program of the Catalan littoral (NW Mediterranean) during a DSP event in 2012. Also in that circumstance, the closure of the production area due to OA levels above the regulatory limit was observed. On the contrary, other studies (Blanco et al., 2017) reported AZA concentrations up to a maximum of 5.4 mg of AZA1 equivalent kg^−1^ in mussels (*M*. *galloprovincialis*) collected during the official monitoring programs of production areas located along the Atlantic and Cantabrian coasts of Spain. The authors supposed that this contamination was linked to the downwelling or upwelling relaxation in the outer (more oceanic) part of the sampling zones, and the molluscs became affected when the plankton populations were advected to the shore.

### Validation study

The method was validated for the criteria established in Regulation (European Community, 2004c) No 882/2004. The linearity was tested by five points calibration curves in the range 1.5–50 μg l^−1^ for OA-group, 1.5–40 μg l^−1^ for AZA- and PTX-group, and 3.75–250 μg l^−1^ for YTX-group. The correlation coefficient indicated a good fit for all the analytes as reported in Table 6.

**Table 6:** Correlation coefficient for the investigated lipophilic marine biotoxins.

**Biotoxin**	***R*^2^**
OA	0.9993
DTX1	0.9991
DTX2	0.9944
PTX2	0.9996
AZA1	0.9998
AZA2	0.9990
AZA3	0.9998
YTX	0.9992
1-Homo YTX	0.9997

The specificity was tested by analyzing 20 blank samples of different mollusc species (mussels, clams and oysters). All blank samples showed no interfering peaks in the retention time of interest for all the analytes. These blank samples were used to evaluate the limit of detection (LOD) and the limit of quantitation (LOQ) of the method, that resulted below the target concentration established by the EU harmonized SOP (2015). The LOD corresponded to 8 μg kg^−1^ for OA-, PTX-, DTX-, and AZA-group, and to 0.013 mg kg^−1^ for YTX-group, while the LOQ was 40 μg kg^−1^ for OA-, PTX-, DTX-, and AZA-group, and 0.060 mg kg^−1^ for YTX-group.

A negative sample spiked with all biotoxins for six samples at three levels in two different days considering the maximum limits and the TEF to calculate the TEQ (Toxicity Equivalent Quantitation) was used to calculate the recovery. In Tables 7–9 the three selected levels for validation were shown. Repeatability and reproducibility data were reported for each level in Tables 10, 11. The measurement uncertainty (MU) was calculated according to EURACHEM/CITAC Guide CG4 (2012) and shown in Table 12.

**Table 7:** Levels of validation for the determination of Toxicity Equivalent Quantitation for OA-group.

**Biotoxin**	**TEF**	**Level 1 (μg kg^−1^) (*n* = 6)**	**TEQ level 1 (μg kg^−1^)**	**Level 2 (μg kg^−1^) (*n* = 6)**	**TEQ level 2 (μg kg^−1^)**	**Level 3 (μg kg^−1^) (*n* = 6)**	**TEQ level 3 (μg kg^−1^)**
OA	1	45	45	67.5	67.5	90	90
DTX1	1	45	45	67.5	67.5	90	90
DTX2	0.6	45	27	67.5	40.5	90	54
PTX2	1	45	45	67.5	67.5	90	90
Sum (as μg of OA equivalent kg^−1^)			162		243		324

**Table 8:** Levels of validation for the determination of Toxicity Equivalent Quantitation for AZA-group.

**Biotoxin**	**TEF**	**Level 1 (μg kg^−1^) (*n* = 6)**	**TEQ level 1 (μg kg^−1^)**	**Level 2 (μg kg^−1^) (*n* = 6)**	**TEQ level 2 (μg kg^−1^)**	**Level 3 (μg kg^−1^) (*n* = 6)**	**TEQ level 3 (μg kg^−1^)**
AZA1	1	40	40	60	60	80	80
AZA2	1.8	40	72	60	108	80	144
AZA3	1.4	40	56	60	84	80	112
Sum (as μg of AZA equivalent kg^−1^)			168		252		336

**Table 9:** Levels of validation for the determination of Toxicity Equivalent Quantitation for YTX group.

**Biotoxin**	**TEF**	**Level 1 (mg kg^−1^) (*n* = 6)**	**TEQ level 1 (mg kg^−1^)**	**Level 2 (mg kg^−1^) (*n* = 6)**	**TEQ level 2 (mg kg^−1^)**	**Level 3 (mg kg^−1^) (*n* = 6)**	**TEQ level 3 (mg kg^−1^)**
YTX	1	0.94	0.94	1.88	1.88	2.82	2.82
1-Homo YTX	1	0.94	0.94	1.88	1.88	2.82	2.82
Sum (as mg of YTX equivalent kg^−1^)			1.88		3.75		5.64

**Table 10:** Repeatability data.

**Biotoxin**	**First level (*n* = 6)**			**Second level (*n* = 6)**			**Third level (*n* = 6)**		
	**R%**	**SD%**	**CV%**	**R%**	**SD%**	**CV%**	**R%**	**SD%**	**CV%**
OA	92.4	10.0	10.8	94.5	6.7	7.1	86.1	6.9	8.0
DTX1	96.6	7.4	7.7	102.0	10.0	9.8	90.0	9.2	10.2
DTX2	101.6	8.9	8.8	104.6	4.9	4.7	94.9	10.0	10.5
PTX2	93.3	7.0	7.5	100.4	6.2	6.2	93.4	5.5	5.9
AZA1	89.5	7.1	7.9	91.5	4.5	5.2	87.4	3.2	3.6
AZA2	100.4	5.5	5.5	102.1	4.4	4.3	97.7	3.1	3.2
AZA3	89.5	4.7	5.3	84.5	3.5	4.2	88.4	7.4	8.4
YTX	84.7	9.5	11.2	90.6	7.5	8.3	92.6	5.1	5.5
1-Homo YTX	87.4	7.5	8.6	91.4	6.3	6.9	95.3	4.1	4.3

*R, Recovery; SD, Standard Deviation; CV, Coefficient Variation*.

**Table 11:** Reproducibility data.

**Biotoxi n**	**First level (*n* = 12)**			**Second level (*n* = 12)**			**Third level (*n* = 12)**		
	**R%**	**SD%**	**CV%**	**R%**	**SD%**	**CV%**	**R%**	**SD%**	**CV%**
OA	91.0	9.7	10.7	91.6	10.0	10.9	89.0	8.6	9.6
DTX1	96.3	7.1	7.4	102.8	8.1	7.8	96.2	10.1	10.5
DTX2	101.6	10.1	9.9	102.3	7.7	7.6	92.0	4.7	5.1
PTX2	93.7	5.6	6.0	96.5	6.9	7.2	93.4	5.5	5.9
AZA1	89.3	5.8	6.5	90.6	4.4	4.8	88.1	3.1	3.5
AZA2	100.0	6.6	6.6	103.7	4.6	4.4	101.3	5.8	5.7
AZA3	89.0	5.2	5.9	85.7	3.7	4.4	86.3	5.9	6.8
YTX	89.7	9.3	10.4	91.4	6.3	6.9	90.7	5.0	5.5
1-Homo YTX	89.9	6.8	7.5	91.7	7.0	7.6	92.6	4.7	5.1

*R, Recovery; SD, Standard Deviation; CV, Coefficient Variation*.

**Table 12:** Measurement uncertainty (MU).

**Biotoxin**	**MU (%)**
OA	22.8
DTX1	20.7
DTX2	13.0
PTX2	8.4
AZA1	10.8
AZA2	12.7
AZA3	16.1
YTX	5.5
1-Homo YTX	8.4

## Conclusion

The legislation of the European Union is particularly careful about the protection of consumers from biological and chemical hazards such as the presence of contaminants in food. With regards to marine biotoxins, it requires that the sampling is carried out weekly during the period at which harvesting is allowed. The small quantities of LMB found in mussels analyzed in the present study demonstrated the good condition of the investigated marine areas as well as the security of bivalve molluscs for public health. However, when these values exceeded the maximum limits, the closure of the examined aquaculture farms should have a negative impact on the seafood industry. The method applied in this study was able to define both presence and concentrations of LMB, that must be routinely monitored in order to avoid the risk of a chronic exposure in regular consumers.

## Author contributions

MB and GM conceived and designed the experiments; FC and FD performed the experiments; MS, PV, RT, and GS analyzed the data; NF contributed reagents, materials, analysis tools; MS and PV wrote the paper.

### Conflict of interest statement

The authors declare that the research was conducted in the absence of any commercial or financial relationships that could be construed as a potential conflict of interest.

## Footnotes

^1^*Piano Pluriennale Regionale Integrato dei Controlli della Sanità Pubblica veterinaria e Sicurezza Alimentare della Regione Abruzzo 2015–2018*. *3*^*rd*^ *Edn*. 1–892.
